# Preclinical Study of Antineoplastic Sinoporphyrin Sodium-PDT via In Vitro and In Vivo Models

**DOI:** 10.3390/molecules22010112

**Published:** 2017-01-11

**Authors:** Rui Shi, Chao Li, Zhihuan Jiang, Wanfang Li, Aiping Wang, Jinfeng Wei

**Affiliations:** New Drug Safety Evaluation Centre, Institute of Materia Medica, Chinese Academy of Medical Sciences and Peking Union Medical College, Beijing 100050, China; shirui87617@foxmail.com (R.S.); kcasper@imm.ac.cn (C.L.); jnkic@163.com (Z.J.); liwanfang@imm.ac.cn (W.L.); wangaiping@imm.ac.cn (A.W.)

**Keywords:** photodynamic therapy, sinoporphyrin sodium, clonogenic assay, MTT assay, xenografts model

## Abstract

Photodynamic therapy (PDT) investigations have seen stable increases and the development of new photosensitizers is a heated topic. Sinoporphyrin sodium is a new photosensitizer isolated from Photofrin. This article evaluated its anticancer effects by clonogenic assays, MTT assays and xenograft experiments in comparison to Photofrin. The clonogenicity inhibition rates of sinoporphyrin sodium-PDT towards four human cancer cell lines ranged from 85.5% to 94.2% at 0.5 μg/mL under 630 nm irradiation of 30 mW/cm^2^ for 180 s. For MTT assays, the IC_50_ ranges of Photofrin-PDT and sinoporphyrin sodium-PDT towards human cancer cells were 0.3 μg/mL to 5.5 μg/mL and 0.1 μg/mL to 0.8 μg/mL under the same irradiation conditions, respectively. The IC_50_ values of Photofrin-PDT and sinoporphyrin sodium-PDT towards human skin cells, HaCaT, were 10 μg/mL and 1.0 μg/mL, respectively. Esophagus carcinoma and hepatoma xenograft models were established to evaluate the in vivo antineoplastic efficacy. A control group, Photofrin-PDT group (20 mg/kg) and sinoporphyrin sodium group at three doses, 0.5 mg/kg, 1 mg/kg and 2 mg/kg, were set. Mice were injected with photosensitizers 24 h before 60 J 630 nm laser irradiation. The tumor weight inhibition ratio of 2 mg/kg sinoporphyrin sodium-PDT reached approximately 90%. Besides, the tumor growths were significantly slowed down by 2 mg/kg sinoporphyrin sodium-PDT, which was equivalent to 20 mg/kg Photofrin-PDT. In sum, sinoporphyrin sodium-PDT showed great anticancer efficacy and with a smaller dose compared with Photofrin. Further investigations are warranted.

## 1. Introduction

Photodynamic therapy (PDT) has been considered as a promising antineoplastic therapy since the 1990s. This new therapy is based on the specialty of photosensitizers (PS), which can accumulate selectively in tumor tissues and generate free radicals upon laser irradiation at a specific wavelength [1]. Preference localization of the photosensitizer towards tumor tissues and irradiation with a precise laser delivery system guarantee the PDT’s double selectiveness, which improves the antineoplastic efficacy while reducing the toxicity at the same time. In addition, PDT killing is based on free radicals attacking, which is non-specific, indicating that it could be utilized repeatedly without significant resistance being observed.

**Figure 1:**
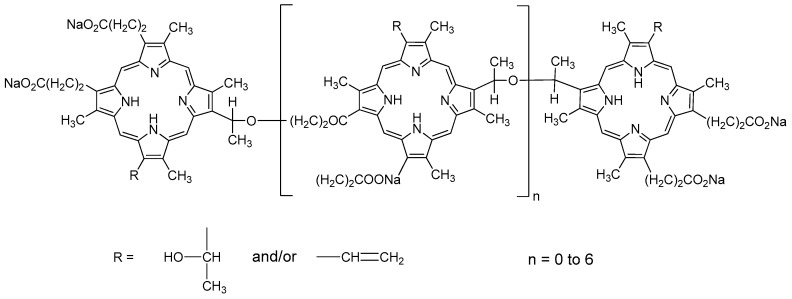
Structure of Photofrin.

**Figure 2:**
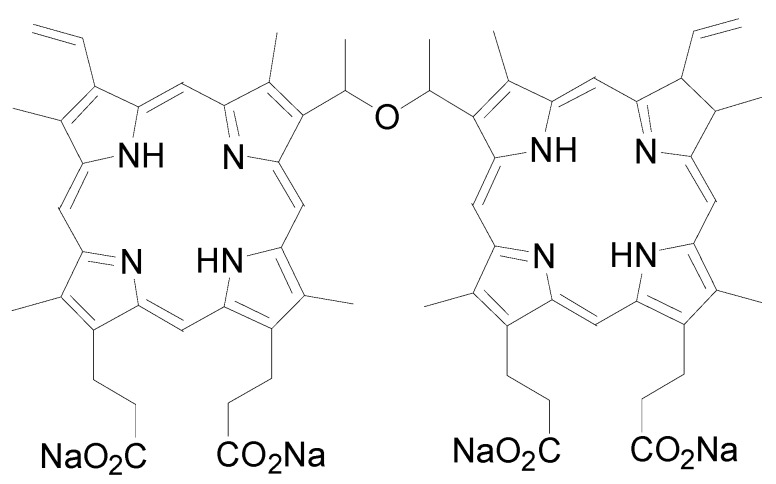
Structure of sinoporphyrin sodium (C_68_H_66_N_8_O_9_Na_4_).

As early as 1988, the porphyrin dimer with an ether linkage has been synthesized from 2-(1-hydroxyethyl) deuteroporphyrin dimethyl ester and found to be an effective antineoplastic photosensitizer in the murine SMT-F tumor model [2]. Since then, more porphyrin dimers have been proved to be effective towards malignant cells in vivo and with reduced skin phototoxicity [3]. Porphyrin-based structure chemicals usually have five absorbance bands in their excitation spectrum. The Soret bond is located at approximately 420 nm and four Q bonds are distributed from 500 nm to 700 nm. Upon illumination, the quantum yields of singlet oxygen are satisfactory in porphyrin derivatives. Sinoporphyrin sodium is a component of Photofrin, the first US Food and Drug Administration (FDA) approved photosensitizer in the world (Figure 1). As a promising anticancer photosensitizer candidate, sinoporphyrin was first isolated from Photofrin and proved to be one of the most potent photoactive compounds (Figure 2). Fang et al. designed the new synthesis route of this compound with high synthetic yield by using the protoporphyrin dimethyl ester as a raw material. The reaction product is separated and purified by a silica gel column. A satisfactory purity can be achieved at 98.5% [4,5]. Its great water solubility and reliable efficacy tested in a pilot screening study make it a good photosensitizer candidate for PDT research [6,7].

This article evaluates sinoporphyrin sodium-mediated photodynamic therapy (sinoporphyrin sodium-PDT) thoroughly via in vitro and in vivo models. Photofrin, the front-line agent in the PDT field, thereby was chosen as the positive control in our study. The clonogenic assay, cell proliferation assay (CPA) and tumor growth evaluation in athymic mice were utilized to evaluate the anticancer efficacy of sinoporphyrin sodium-PDT.

## 2. Results

### 2.1. Clonogenic Assay

**Figure 3:**
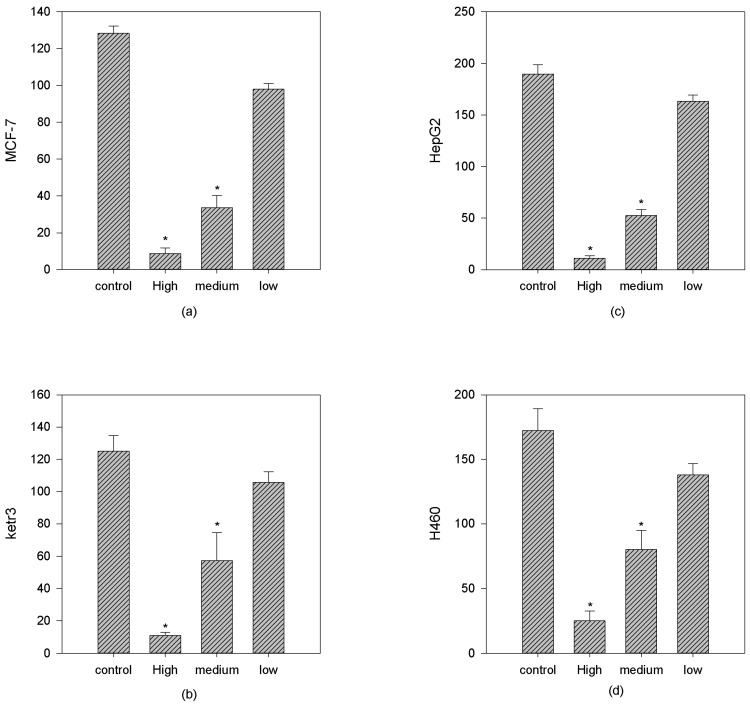
The clonogenic assays of sinoporphyrin sodium-PDT towards human cancer cells. (**a**) Breast adeocarcinoma MCF-7; (**b**) Renal carcinoma ketr3; (**c**) Hepatoma HepG2; (**d**) Large cell lung cancer H460. Control groups (10% FBS-containing complete medium); Sinoporphyrin sodium concentration: high groups (0.5 μg/mL), medium groups (0.05 μg/mL) and low groups (0.005 μg/mL). * *p* < 0.05, when compared with control groups.

For the clonogenic assay, breast adenocarcinoma MCF-7, renal carcinoma Ketr 3, liver hepatoma HepG2 and large cell lung cancer H460 cell lines were explored at three dose levels of sinoporphyrin sodium, which were 0.5 μg/mL, 0.05 μg/mL, and 0.005 μg/mL, respectively, and received identical doses of light illumination (Figure 3).

The clonogenicity inhibition rates increased in a photosensitizer dose-dependent manner (Table 1). The highest dose plus laser illumination could inhibit clone viabilities of all four tumor cell lines by 85% to 94%.

### 2.2. Cell Viability Assay

Eleven human cancer cell lines as well as normal human skin cells, human immortal keratinocyte cells, HaCaT, were explored in the cell viability assay. The IC_50_ values for both the positive control Photofrin and sinoporphyrin sodium were achieved for each cell line (Table 2). For human cancer cell lines, the IC_50_ ranges of Photofrin and sinoporphyrin sodium were from 0.3 μg/mL to 5 μg/mL and 0.1 μg/mL to 1 μg/mL, respectively. For human immortal keratinocyte cells, the IC_50_ values of Photofrin^®^ and sinoporphyrin sodium were approximately 10.4 μg/mL and 1.4 μg/mL, respectively.

### 2.3. Sinoporphyrin Sodium-PDT Antineoplastic Efficacy Evaluation

#### 2.3.1. Human Esophagus Tumor CaEs-17 Xenograft Models

**Figure 4:**
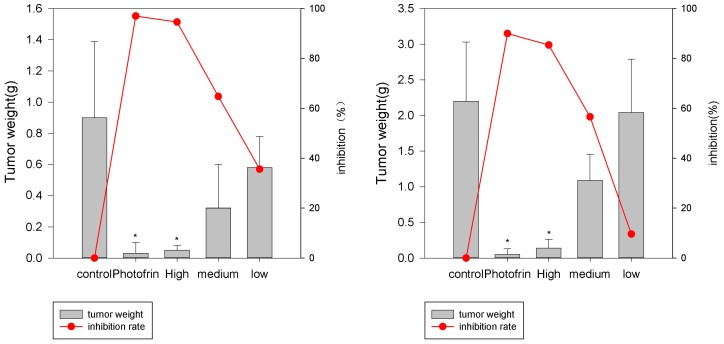
Tumor weights in esophagus tumor CaEs-17 xenografts. First experiment (**left**); second experiment (**right**). Control (0.9% NaCl), Photofrin (20 mg/kg); Sinoporphyrin sodium concentration: high dose groups (2 mg/kg), medium dose groups (1 mg/kg), and low dose groups (0.5 mg/kg). * *p* < 0.05, when compared with control groups.

There were two aspects of the xenograft model experiments: the tumor weights and the tumor volumes. The tumor weights in all groups are shown in the bar graphs in Figure 4, which possess a clear sinoporphyrin sodium dose-dependent manner in the three treatment groups. The tumor weight inhibition ratios are plotted as the red full line in Figure 4. The 2 mg/kg sinoporphyrin sodium-PDT had a similar anticancer efficacy as 20 mg/kg Photofrin-PDT.

The tumor volumes of xenografts in tumor-bearing mice were measured continuously during the experiments. The tumor volumes of all groups at the beginning and end of the experiments are listed in Table 3. The relative tumor volume (RTV) and ratio of RTV_treatment_ and RTV_control_ (T/C) were also calculated.

#### 2.3.2. Human Hepatoma HepG2

**Figure 5:**
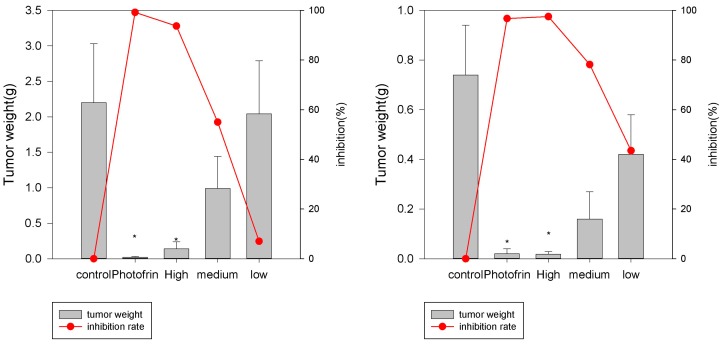
Tumor weights in hepatoma HepG2 xenografts. First experiment (**left**); second experiment (**right**). Control (0.9% NaCl), Photofrin (20 mg/kg); Sinoporphyrin sodium concentration: high dose groups (2 mg/kg), medium dose groups (1 mg/kg), and low dose groups (0.5 mg/kg). * *p* < 0.05, when compared with control groups.

The tumor weights in all groups as well as the inhibition rates are plotted as bar graphs and red full lines in Figure 5. The similar inhibition trends shown in CaEs-17 xenografts were also seen in Hepatoma HepG2 xenograft experiments.

The tumor volumes of xenografts in tumor-bearing mice were measured continuously during the experiments. The tumor volumes of all groups and the RTV and T/C values in hepatoma HepG2 experiments are shown in Table 4.

## 3. Discussion

Photodynamic therapy in oncology has been a heated topic in the PDT field, since the mainstream regimens could not fully live up to the high expectations of the patients. The dual selectivity achieved by the preferential photosensitizer uptake of malignant cells and the targeted laser illumination enable PDT to be utilized for both curative and alleviative purposes. The comparatively transient and tolerable adverse effects profiles make this treatment a good alternative regimen that can be considered when standard treatment fails or cannot be tolerated.

Preclinical investigation is crucially important in the oncology field given the high failure rates and huge investments into subsequent clinical trials. The traditional clonogenic assay, the MTT assay as well as xenograft models are still the most used screening tests in evaluating anticancer candidates [8].

The clonogenic assays examined the effect of sinoporphyrin sodium-PDT towards the clonogenicity ability of MCF-7, H460, HepG2 and ketr 3 cell lines at different dose levels and with identical laser illumination. Decreased clone survivals were observed in all treatment groups. At the 0.5 μg/mL dose level, few clones were observed in all cell lines. At the 0.05 μg/mL dose level, the clonogenic ability varied within the four cell lines, ranging from 50% to 80%.

In the cell viability assay, we tested 11 human cells lines, which included hepatoma, colorectal cancer, breast cancer, lung cancer, melanoma, ovarian cancer, neuronal glioblastoma, renal carcinoma as well as gastric cancer. These cancer types are among the most fatal diseases throughout the world, with high incidence and high mortality at the same time. According to the results, the cancer cells showed different levels of responsiveness to the two PDT regimens. Generally, colorectal cancer and breast cancer were more responsive to both Photofrin-PDT and sinoporphyrin sodium-PDT. The results indicated that Photofrin-PDT exhibited great antineoplastic efficacy towards multiple cancer cell lines with IC_50_ values ranging from 0.3 μg/mL to 5.5 μg/mL. Meanwhile, the IC_50_ values of sinoporphyrin sodium-PDT were 0.1 μg/mL to 0.8 μg/mL. For normal cells, the killing efficacy of PDT mediated by both photosensitizers was decreased given that the IC_50_ values of Photofrin-PDT and sinoporphyrin sodium-PDT were 10 times higher towards the human immortal keratinocyte HaCaT cell line.

In the esophagus tumor and hepatoma xenograft experiments, the sinoporphyrin sodium’s dose-dependent killing manner was clearly observed. In terms of tumor volume, the PDT treatment obviously slowed down the tumor growth in both models. Interestingly, in the 2 mg/kg sinoporphyrin sodium-PDT and 20 mg/kg Photofrin-PDT groups, the tumor shrinkages were also observed, which indicated that PDT treatment not only prevents tumor proliferation, but also exerts effects on the tumor mass that had been inoculated before the PDT process. It was highly possible that other mechanisms, such as anti-vasculature as well as autophagy pathways, may exist in the experiments [9].

Further, the phototoxicities observed in the in vivo experiments were also of importance. The skin toxicity had not been quantitatively evaluated in this article. In our experiments, only acute skin toxicity was observed, which appeared the next day after PDT irradiation at the illumination site and neighboring areas of mice with the presentation of swelling and edema. The whitened color indicated impaired blood vasculature which lasted for the following three to four days. The incrustation took place approximately at days 4–5 after irradiation. The color of the scab in the Photofrin group was yellow and that of the sinoporphyrin sodium group was white. The duration of swelling varied with different treatment groups. Swelling of the illumination spots continued for seven to 10 days for animals in the Photofrin and high sinoporphyrin sodium dose group. After the swelling retreated, the tumor sites in the treatment groups tended to be flat and began to shrink. The local injuries after PDT irradiation were described in one study which showed that severe hypoxia was the main physiological change in the tissues [10]. This observation indicated that PDT at the high dose level may cause acute and severe phototoxicity. No persistent skin toxicity has been observed under indoor light illumination in our experiments.

Many chemical structures have been developed as photosensitizer candidates, such as porphyrins, chlorins and phthalocyanines. The phthalocyanine and chlorin compounds usually have longer excitation wavelengths and stronger absorbance ensuring deeper penetration into the tissue, which may further expand the cancer profiles that PDT could treat. However, for porphyrin derivatives, the comparatively shorter excitation wavelength and thereby the superficial illumination area are now difficult to improve. However, given their good safety profile, porphyrins are still the most used photosensitizers in clinical settings.

Researchers have been investigating ways to improve porphyrins-PDT, such as with the development of optical fibers coupled with PDT as well as structure modification of the photosensitizers to increase their targeting efficacy [11]. Besides, designing reasonable PDT regimens based on pharmacokinetic parameters of photosensitizers could also achieve this goal. Xiong W. et al. [12] conducted a comparative study of two kinds of repeated sinoporphyrin sodium-PDT strategies using the breast cancer xenograft model. In regimen 1, sinoporphyrin sodium was only injected one time and then the mice were exposed to the 50 J/cm^2^ laser 24 h, 30 h and 36 h after photosensitizer administration. In regimen 2, sinoporphyrin sodium was injected three times and mice received the 50 J/cm^2^ laser 24 h after each injection. The tumor volume inhibition ratios were 85.8% ± 7.6% and 65.7% ± 8.6% for regimen 1 and 2, respectively. Also, Kessel D. [13] explored the possibility of a two-sensitizer sequential PDT protocol to enhance the efficacy of photo-killing. The possible mechanism was that the photo-damage caused by a low-level lysosome-targeted photosensitizer could promote the apoptotic responses caused by a subsequent mitochondria-targeted photosensitizer. These two methods were new but reasonable ways to optimize PDT treatment strategies based on photosensitizers’ pharmacodynamic and pharmacokinetic profiles.

Wang H. et al. [14] explored the cellular uptake of sinoporphyrin sodium in ECA-109 cells and found it was mainly located in the mitochondria. Given the short existence time and thereby the short diffusion distance of reactive oxygen species (ROS), the cytotoxicity mechanism of sinoporphyrin sodium-mediated PDT may be induced by injury to the mitochondria. Injury to the mitochondria may initiate apoptotic responses, including cytochrome c release into the cytoplasm and caspase protein activation [15]. Lv W. et al. [16] found that the efficacy of a mitochondria-targeted photosensitizer-mediated PDT may be improved under a hypoxic environment, which may be another way to improve sinoporphyrin sodium-PDT.

There were some limitations of this research. First, the traditional subcutaneous implanted xenograft models we used in the sinoporphyrin sodium-PDT evaluation may be different from the natural pathogenesis of esophagus and hepatocellular carcinoma compared with orthotopic transplantation models and transgenic or knock-out animals [17]. We finally chose subcutaneously inoculated models (s.c. models), which usually do not have lymphatic and distal metastasis, given that PDT has been mostly utilized in premalignant and early stages of cancer therapy. So, it should be noted that s.c. inoculated models were used in this study when the results are to be interpreted and compared. Second, the point-to-point laser illumination mode avoided many possible adverse effects that could take place in the clinic, such as bleeding, esophageal fistula, etc., introduced by an endoscopic fiber-coupled PDT process [18]. Third, we did not explore the possible different laser parameters that could further improve the efficacy.

## 4. Materials and Methods

### 4.1. Monolayer Cell Culture

Neuronal glioblastoma U251, hepatoma bel7402, breast adeocarcinoma MCF-7, gastric carcinoma BGC-823, renal carcinoma ketr 3, ovarian carcinoma A2780, colorectal carcinoma HCT-8, colon carcinoma HCT-116, hepatoma HepG2, malignant melanoma A375, large cell lung cancer NCI-H460 (Institute of Materia Medica, Chinese Academy of Medical Science & Peking Union Medical College, CAMS & PUMC, Beijing, China) were routinely kept and passaged in our laboratory. Immortal human keratinocyte HaCaT was purchased from Cell Culture Center, Institute of Basics Medical Sciences, CAMS & PUMC). All cell lines were cultured in appropriate culture medium, RP1640, DMEM or MEM medium containing 10% fetal calf serum (CBS, Zhejiang Tianhang Biotechnology Co., Ltd., Hangzhou, China) in 5% CO_2_ incubator at 37 °C. Cells were passaged regularly every three to four days with 0.05% trypsin (Hyclone, Logan, UT, USA). Trypsin was added to the cell cultures following a 20 min incubation period with phosphate-buffered saline (Hyclone, Logan, UT, USA).

### 4.2. Clonogenic Assay

The sinoporphyrin sodium (bis[1-[6,7-bis[2-(sodium carbonate) ethyl] 1,3,5,8-tetra-methyl-2-vinyl-porphin-4-yl]-ethyl] ether, Qinglong High-Tech Co., Ltd., Yichun, China, Figure 1) stock solution was prepared in 0.9% NaCl, which at a concentration of 2 mg/mL. Further dilution was performed in 10% FCS (Zhejiang Tianhang Biotechnology Co., Ltd., Hangzhou, China) RPMI1640 media (Gibco^®^, Grand Island, NY, USA). Three working solution was achieved at the final concentration of 0.5 μg/mL, 0.05 μg/mL, 0.005 μg/mL, respectively. Four groups were set in clonogenic assay (1) control group; (2) Low dose group with 0.005 μg/mL sinoporphyrin sodium; (3) Medium dose group with 0.05 μg/mL sinoporphyrin sodium; and (4) High dose group with 0.5 μg/mL sinoporphyrin sodium. All groups were done in triplicate.

MCF-7, ketr 3, HepG2 and H460 cells were seeded in six-well plates (Corning, NY, USA) at density of 200 cells per well and incubated under condition of 37 °C and 5% CO_2_ (Thermo, Waltham, MA, USA) for 24 h. The cells were rinsed with plain medium and then dosed with sinoporphyrin sodium at 0.005 μg/mL, 0.05 μg/mL and 0.5 μg/mL. The cells in control well were incubated with complete medium. Four hours after dosing, the medium in all plates were replaced with plain media and were about to undergo PDT process. The 630 nm laser (Xingda Photoelectric Medical Instrument Co., Guilin, China) was applied to incubation well at fluence rate of 30 mW/cm^2^ and lasted for 180 s well by well. The total illumination power was 5.4 Joules per well. Plain medium in all wells were replaced with 10% FBS-containing complete medium after PDT process. And, all plates were returned to incubator for the next seven days. After the incubation period, the medium were discarded and cells were rinse twice with 2 mL PBS (Hyclone, Logan, UT, USA). The cells were fixed with 2 mL methanol (Chemical Reagent Beijing Co., Ltd., Beijing, China) for 5 min. This process was repeated twice. Then, the cells were stained with 2 mL 0.1% violet solution (Chemical Reagent Beijing Co., Ltd., Beijing, China) for 5 min. The staining solution was washed away by pure water. After fully drying, the clones in each plate were counted by naked eyes. The clone numbers in triplicate were averaged and compared with control group. The Clonogenicity efficiency (CE%, Equation (1)) and clonogenicity inhibition rate (CI%, Equation (2)) were calculated.

(1) $$\mathrm{CE}\%=\frac{N}{N_{0}}\times 100\%,$$

(2) $$\mathrm{CI}\%=(1-\frac{N_{\mathrm{treatment}}}{N_{\mathrm{control}}})\times 100\%,$$

where:

(1) CE% stands for clonogenicity efficiency percentage
(2) CI% stands for clonogenicity inhibition percentage
(3) N was clone number of each group. N_0_ was cells seeded in well at day 1, which was 200 in this case.
(4) N_treatment_ was clone number of each treatment group. N_control_ was clone number of control group.

### 4.3. Monolayer PDT Efficacy via MTT Assay

**Figure 6:**
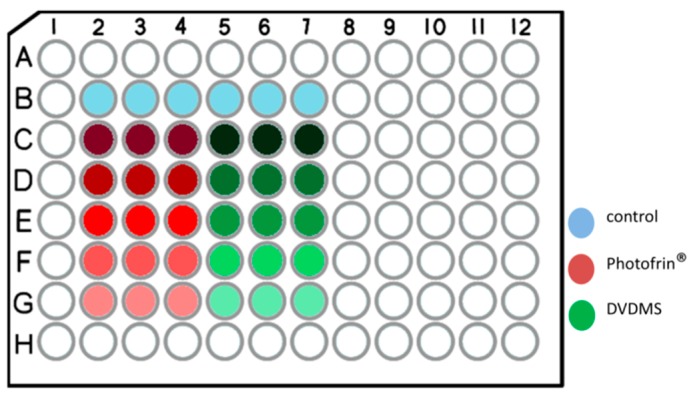
The 96-well plate configuration of MTT assay. Plate configuration: Blue, Row B from B2 to B7 was control groups, which were incubated with 0.9% NaCl. Red panel indicated positive control of Photofrin^®^: Row C, from C2 to C4, concentration = 100 μg/mL; Row D, from D2 to D4, concentration = 10 μg/mL; Row E, from E2 to E4, concentration = 1 μg/mL; Row F, from F2 to F4, concentration = 0.1 μg/mL; Row G, from G2 to G4, concentration = 0.01 μg/mL; Green panel indicated sinoporphyrin sodium (DVDMS): Row C, from C5 to C7, concentration = 10 μg/mL; Row D, from D5 to D7, concentration = 1 μg/mL; Row E, from E5 to E7, concentration = 0.1 μg/mL; Row F, from F5 to F7, concentration = 0.01 μg/mL; Row G, from G5 to G7, concentration = 0.001 μg/mL.

Monolayer response to Sinoporphyrin sodium-PDT and Photofrin-PDT were measured using the MTT colorimetric assay. Human cancer cell lines of U251, bel7402, MCF-7, BGC-823, ketr 3, A2780, HCT-8, HCT-116, HepG2, A375 as well as normal human cell HaCaT were included in MTT assays. The figuration of 96-well plate was divided into positive control part and sinoporphyrin sodium part (Figure 6).

Single cell solution were made and seeded in 96-well plates (Corning, NY, USA) at density of 1000 cells per well 24 h prior to experiment. Triplicates were done for each cell line. The 96-well plates were incubated under condition of 37 °C and 5% CO_2_ (Thermo, Waltham, MA, USA). The cells were dosed with different concentration of Photofrin^®^ (AXCAN PHARMA INC, Lot OK 102, Mont Saint Hilaire, QC, Canada) or Sinoporphyrin sodium after PBS rinse. The concentration set in Photofrin^®^ part were 100 μg/mL, 10 μg/mL, 1 μg/mL, 0.1 μg/mL and 0.01 μg/mL. The concentration in sinoporphyrin sodium part was 10 μg/mL, 1 μg/mL, 0.1 μg/mL, 0.01 μg/mL and 0.001 μg/mL. The cells were co-incubated with two different photosensitizers for subsequent 4 h. Before PDT process, the photosensitizer-containing media were replaced by plain media in all wells. The 630 nm laser (Xingda Photoelectric Medical Instrument Co., Guilin, China) was applied to each plate at fluence rate of 30 mw/cm^2^ with illumination diameter of 80 mm. The illumination lasted for 180 s for each plate. Thus, the total laser power was 1.6 Joules per well. The photosensitizer containing medium was replaced with plain media and the plates were incubated for following 72 h. After three-day incubation, cells were incubated with 150 μL 0.5 mg/mL MTT (Sigma-Aldrich, St. Louis, MO, USA) working solution for 4 h in incubator. Formazan reduced by mitochondria electronic chain of live cells could be seen at the bottom of the well. The 190 μL DMSO (Chemical Reagent Beijing Co., Ltd., Beijing, China) was added into wells to dissolve the formazan for 20 min after supernatant were removed. The optical density values (OD values) of each well were collected by microplate reader (Sunnyvale, CA, USA). The inhibition rates of different dose level of two photosensitizers were calculated (Equation (3)). The IC_50_ values were achieved by plotting the values against the concentration.

(3) $$\mathrm{Cell}\;\mathrm{Proliferation}\;\mathrm{inhibition}\;\mathrm{rate}=(1-\frac{{\mathrm{OD}}_{\mathrm{treatment}}}{{\mathrm{OD}}_{\mathrm{control}}})\times 100\%,$$

### 4.4. Establishing Xenograft Models

The xenograft-bearing nude mice were established to evaluate the efficacy of sinoporphyrin sodium-PDT. Xenografts of human esophagus carcinoma CaEs-17 and hepatoma HepG2 were kindly donated by Professor Zhaodi Fu (Institute of Materia Medica, CAMS & PUMC). The xenografts of esophagus carcinoma CaEs-17 and hepatoma HepG2 were thawed and passaged in nude mice for two passages. Tumor mass with volume of approximately 1 mm^3^ were inoculated in the right forelimbs of the SPF level Balb/C-nu/nu female mice (Vital River Laboratories, Beijing, China), weighing 18 to 20 g. After inoculation, mice were bred in SPF level animal hood under controlled temperature and humidity. Food and water were provided ad libitum. The major and minor diameters of implants were measured by caliper every two days until the tumor volume were about 100 mm^3^ (Equation (4)).

(4) $$\mathrm{Tumor}\;\mathrm{Volume}\;(\mathrm{TV})=\frac{D_{\mathrm{length}}\times {D_{\mathrm{width}}}^{2}}{2},$$

### 4.5. Antineoplastic Efficacy Evaluation via Xenograft Models

Five groups, including control group (solvent control), positive control (Photofrin^®^ 20 mg·kg^−1^) and sinoporphyrin sodium low dose group (0.5 mg·kg^−1^), medium dose group (1 mg·kg^−1^) and high dose group (2 mg·kg^−1^) were set in the experiments. The photosensitizers were administered through tail vein when the tumor volumes of xenografts in all groups reached approximately 100 mm^3^. Except control group, all treatment groups received identical laser illumination at 127.7 mW/cm^2^ 24 h after photosensitizer administration. The laser beam was aimed at tumor inoculation area within a diameter of 10 mm and lasted for 600 s. The total laser power was 60 Joules per animal. Then all mice were kept under SPF level animal facility for next 21 days, during which time, the tumor sizes were monitored by caliper measurement. At the end of recovery period, animal body and tumor weight were measured and animals were sacrificed. The tumor were then dissected out and weighed. The relative tumor volume (RTV, Equation (5)) values and T/C values (Equation (6)) were calculated and compared among groups. Both xenografts experiments were conducted twice to ensure the accuracy.

(5) Relative tumor volume (RTV)=VtV0,

(6) $$T/C\;(\%)=\frac{{\mathrm{RTV}}_{\mathrm{treatment}}}{{\mathrm{RTV}}_{\mathrm{control}}}\times 100\%,$$

where:

(1) V_t_ stands for tumor volume at different days in the treatment periods.
(2) V_0_ stands for the tumor volume at the day 1 of the experiment.
(3) RTV_treatment_ stands for RTV values of different treatment groups.
(4) RTV_control_ stands for RTV value of control group.

The experiment design and procedures involving laboratory animals were approved by Institutional Animal Care and Use Committee (IACUC) in Institute of Materia Medica, CAMS & PUMC. All animal manipulation was complied with the Association for Assessment and Accreditation of Laboratory Animal Care (AAALAC) guidelines for animal welfare.

### 4.6. Data Process and Statistical Study

All numerical data were expressed as mean ± SD and were statistically compared by One-Way ANOVA using software SPSS 16.0 (Chicago, IL, USA). The *p* values < 0.5 was accepted as significance.

## 5. Conclusions

The present study showed that sinoporphyrin sodium-PDT possessed a great antineoplastic effect towards both monolayer human cancer cells and xenograft models compared with Photofrin. Given the lesser dose used and the safety profile observed in our studies, sinoporphyrin sodium was proved to be a good photosensitizer candidate for use in the oncology field. Further studies are warranted.

## Figures and Tables

**Table 1:** The clonogenicity inhibition rates of sinoporphyrin sodium-PDT towards human cancer cells (%). MCF-7, breast adenocarcinoma; Ketr 3, renal carcinoma; HepG2, liver hepatoma; H460, large cell lung cancer.

Sinoporphyrin Concentration (μg/mL)	Clonogenicity Inhibition Percentage (%)			
	MCF-7	Ketr3	HepG2	H460
0.5 μg/mL	93.3 ± 2.2	91.2 ± 1.2	94.2 ± 1.1	85.2 ± 5.9
0.05 μg/mL	73.7 ± 5.9	53.3 ± 17.2	72.2 ± 1.8	53.8 ± 12.6
0.005 μg/mL	23.6 ± 0.4	15.3 ± 5.1	13.7 ± 5.6	19.5 ± 7.7

**Table 2:** IC_50_ values of Photofrin-PDT and sinoporphyrin sodium-PDT towards human cancer cell lines and immortal keratinocyte cells (X ± SD).

Cell Line	IC_50_ (μg/mL) Photofrin^®^	IC_50_ (μg/mL) Sinoporphyrin Sodium
U251	4.5 ± 1.0	0.5 ± 0.2
HaCaT	10 ± 1.0	1.4 ± 0.4
BGC-823	1.5 ± 0.3	0.2 ± 0.1
HepG2	1.4 ± 0.2	0.3 ± 0.2
MCF-7	0.7 ± 0.1	0.08 ± 0.03
H460	3.3 ± 0.9	0.6 ± 0.2
ketr3	2.3 ± 1.0	0.2 ± 0.1
A375	1.9 ± 0.2	0.21 ± 0.01
A2780	1.3 ± 0.3	0.12 ± 0.04
HCT-8	1.4 ± 0.7	0.09 ± 0.04
HCT-116	0.3 ± 0.1	0.09 ± 0.02
Bel7402	2.9 ± 0.4	0.8 ± 0.2

**Table 3:** Tumor volumes, RTV and T/C values in esophagus tumor CaEs-17 xenograft experiments.

Study	Groups		Control	Photofrin	Sinoporphyrin Sodium		
				20 mg/kg	0.5 mg/kg	1 mg/kg	2 mg/kg
First	Tumor volume	Start	98 ± 24	102 ± 24	99 ± 13	102 ± 11	100 ± 13
		End	855 ± 589	15 ± 4	417 ± 190	205 ± 180	23 ± 12
	RTV		8.8	0.2	4.2	2.0	0.2
	T/C		-	1.7	48.0	23.0	2.6
Second	Tumor volume	Start	92 ± 16	97 ± 13	93 ± 18	96 ± 18	93 ± 21
		End	1480 ± 332	19 ± 12	1115 ± 646	599 ± 204	29 ± 21
	RTV		16.0	0.2	12.0	6.2	0.3
	T/C		-	1.2	74.8	39.0	1.9

**Table 4:** Tumor volumes, RTV and T/C values in hepatoma HepG2 xenograft experiments.

Study	Groups		Control	Photofrin	Sinoporphyrin Sodium		
				20 mg/kg	0.5 mg/kg	1 mg/kg	2 mg/kg
First	Tumor volume	Start	80 ± 26	79 ± 32	82 ± 35	82 ± 37	81 ± 35
		End	563 ± 283	9 ± 5	359 ± 103	74 ± 57	22 ± 14
	RTV		7.0	0.1	4.4	0.9	0.3
	T/C		-	1.6	62.6	12.9	3.9
Second	Tumor volume	Start	81 ± 62	84 ± 49	81 ± 39	81 ± 28	82 ± 27
		End	659 ± 280	13 ± 8	303 ± 142	114 ± 80	11 ± 6
	RTV		8.1	0.2	3.8	1.4	0.1
	T/C		-	1.8	46.3	17.3	1.6

## References

[1] Allison R.R., Mota H.C., Sibata C.H. (2004). Clinical PD/PDT in North America: An historical review. Photodiagn. Photodyn. Ther..

[2] Pandey R.K., Dougherty T.J. (1988). Synthesis and photosensitizing activity of a di-porphyrin ether. Photochem. Photobiol..

[3] Pandey R.K., Smith K.M., Dougherty T.J. (1990). Porphyrin dimers as photosensitizers in photodynamic therapy. J. Med. Chem..

[4] Fang Q.C. (2014). Photodynamic therapy for cancer treatment and the new antitumor photosensitizer sinoporphyrin sodium. Chin. J. New Drugs.

[5] Fang Q.C., Yang D. (2012). Ether-Linked Porphyrin Dimer Salts and Their Manufacturing Method.

[6] Jiang Z.H., Shi R., Li C., Wang A.P. (2013). Inhibitory effects of DVDMS-2-based-photodynamic therapy on the growth of tumor in vitro and in vivo. Carcinog. Teratog. Mutag..

[7] Yan X., Niu G., Lin J., Jin A.J., Hu H., Tang Y., Zhang Y., Wu A., Lu J., Zhng S. (2015). Enhanced fluorescence imaging guided photodynamic therapy of sinoporphyrin sodium loaded graphene oxide. Biomaterials.

[8] Buch K., Peters T., Nawroth T., Sänger M., Schmidberger H., Langguth P. (2012). Determination of cell survival after irradiation via clonogenic assay versus multiple MTT Assay—A comparative study. Radiat. Oncol..

[9] Kessel D., Evans C.L. (2016). Promotion of Proapoptotic Signals by Lysosomal Photodamage: Mechanistic Aspects and Influence of Autophagy. Photochem. Photobiol..

[10] Busch T.M. (2006). Local physiological changes during photodynamic therapy. Lasers Surg. Med..

[11] Garland M.J., Cassidy C.M., Woolfson D., Donnelly R.F. (2009). Designing photosensitizers for photodynamic therapy: Strategies, challenges and promising developments. Future Med. Chem..

[12] Xiong W., Wang X., Hu J., Liu Y., Liu Q., Wang P. (2016). Comparative study of two kinds of repeated photodynamic therapy strategies in breast cancer by using a sensitizer, sinoporphyrin sodium. J. Photochem. Photobiol. B Biol..

[13] Kessel D. (2016). Photodynamic therapy: Promotion of efficacy by a sequential protocol. J. Porphyr. Phthalocyanines.

[14] Wang H., Wang X., Zhang S., Wang P., Zhang K., Liu Q. (2014). Sinoporphyrin sodium, a novel sensitizer, triggers mitochondrial-dependent apoptosis in ECA-109 cells via production of reactive oxygen species. Int. J. Nanomed..

[15] Ito H., Matsui H. (2016). Mitochondrial Reactive Oxygen Species and Photodynamic Therapy. Laser Ther..

[16] Lv W., Zhang Z., Zhang K.Y., Yang H., Liu S., Xu A., Song G., Zhao Q., Huang W. (2016). A Mitochondria-Targeted Photosensitizer Showing Improved Photodynamic Therapy Effects under Hypoxia. Angew. Chem. Int. Ed..

[17] Kelland L.R. (2004). Of mice and men: Values and liabilities of the athymic nude mouse model in anticancer drug development. Eur. J. Cancer.

[18] Qumseya B.J., David W., Wolfsen H.C. (2013). Photodynamic Therapy for Barrett’s Esophagus and Esophageal Carcinoma. Clin. Endosc..

